# Experience-based utility and own health state valuation for a health state classification system: why and how to do it

**DOI:** 10.1007/s10198-017-0931-5

**Published:** 2017-10-11

**Authors:** John Brazier, Donna Rowen, Milad Karimi, Tessa Peasgood, Aki Tsuchiya, Julie Ratcliffe

**Affiliations:** 1 School of Health and Related Research (ScHARR), University of Sheffield, Sheffield, UK; 2 Erasmus School of Health Policy & Management, Erasmus University Rotterdam, Rotterdam, The Netherlands; 3 Department of Economics, University of Sheffield, Sheffield, UK; 4 Institute for Choice, School of Business, University of South Australia, Adelaide, Australia

**Keywords:** Experience-based utility, Own health state valuation, Hypothetical health state values, Informed preferences, I10

## Abstract

In the estimation of population value sets for health state classification systems such as the EuroQOL five dimensions questionnaire (EQ-5D), there is increasing interest in asking respondents to value their own health state, sometimes referred to as “experience-based utility values” or, more correctly, own rather than hypothetical health states. Own health state values differ to hypothetical health state values, and this may be attributable to many reasons. This paper critically examines whose values matter; why there is a difference between own and hypothetical values; how to measure own health state values; and why to use own health state values. Finally, the paper examines other ways that own health state values can be taken into account, such as including the use of informed general population preferences that may better take into account experience-based values.

## Introduction

Health state utility values can be obtained from multiple sources, including patients, carers, health professionals, and the community (see [1] for a detailed overview). Currently, health state value sets are usually obtained from members of the general public who attempt to imagine what the state would be like, mainly argued for on the basis that the general population are the payers of healthcare. However, it has also been argued that values should be obtained from patients, as they better understand what it is like to live in poorer health [2–4]. Of course, utility values can be obtained directly from people whether or not they are patients. Own health state valuation, such as the EQ-5D, can be used to estimate value sets for (generic) measures of health or to value a person’s state without a descriptive system. The former is a more recent development and is now being described in the literature as “experience-based utility values,” where respondents own current health state values are modelled against their self-reported EuroQOL five dimensions questionnaire (EQ-5D) health state, e.g., which is used to estimate a value set for all health states described by the EQ-5D [4–7].

The aim of the paper was to critically examine issues in the elicitation and use of experience-based utility values or own health state values and to propose some potential ways forward. We critically examined a number of important issues: whose values matter; why there is a difference between own and hypothetical values; how to measure own health state values; why to use own health state values. Finally, the paper examines other ways that experience can be taken into account, such as the use of informed general population preferences. This paper is based on the opinions of the authors and not a systematic review of the literature, though we draw on our knowledge of the literature.

### A clarification: Whose values and what to value?

There are two main considerations in this literature (Table 1): whose values and what to value. The debate has tended to focus on the first of these: whether to use patient or general public values. The second is whether the public should value their own state or some hypothetical state. Conventionally, health state classification systems such as the EQ-5D are valued using a large valuation survey of the general population. Each respondent is asked to value several hypothetical health states, which are modelled using regression analysis to generate preference weights that enable estimation of a utility value for every health state defined by the classification system (cell 1). An alternative approach is to ask patients to value their own current state, referred to as own health state valuation (cell 4). Patients have also been asked to value hypothetical states (cell 2) and the recent growing interest in experience-based utility values (cell 3). Of course, patients are also members of the general public, and the strict public vs patient dichotomy is a false—albeit informative—one [8]. A typical study in cell 4 usually has a relatively small, clinically homogeneous sample and therefore cannot elicit values for enough states to model the entire descriptive system. A study in cell 3 aims for a large, heterogeneous sample, with the aim to value all possible states in a descriptive system. However, the established literature compares cells 1 and 4 rather than 1 against 3. A third consideration is whether to link values from any of these cells to a descriptive system, e.g., EQ-5D, which is a comparatively recent development in the literature. In common with much of the literature, the term patient is often used in this paper for simplicity to refer to experience-based values, though it is not that the person is a patient that matters per se but their experience of the state.

**Table 1:** Categorizing values by population and what is valued

	General public	Patients
Hypothetical states	Cell 1	Cell 2
Own current state	Cell 3	Cell 4

## How to measure experience-based utility?

The terminology originated with Kahneman [9], who distinguished between (stated) preference-based methods of valuation, such as willingness to pay, standard gamble (SG), and time tradeoff (TTO), which elicit what he calls decision utility, and the hedonic and affective experiential methods associated with an outcome he calls experienced utility. He and others have questioned the validity of decision-utility-based methods due to systematic errors in forecasts of how we may feel in future states [10]. In its place, they argued for a return to an interpretation of utility used by Bentham, in which utility is the pleasure and pain experienced in each moment of time [11]. This would imply a return to measuring utility directly and in cardinal form from the person experiencing it and hence experienced utility [12].

Kahneman recommends measuring utility using moment-based happiness [9]. The most direct method of doing so is the experienced sampling methodology’ [13] in which participants are contacted at multiple random points during the day and asked about how they are feeling and how happy they are at each time point. A more pragmatic and less intrusive solution is the day reconstruction method [14], which asks people to recount different episodes of the previous day and how they felt during each episode. Data on affective experience from the previous day is then combined: one possibility is to take the difference between the average positive feelings (or the most intense positive) and the average negative (or the most intense negative) [14] and estimate an area under the plotted curve (AUC) to define experienced utility over time. Another possibility is to use the proportion of time in which the most intense negative affect outweighs the most intense positive, referred to as a U-index [15].

The methods for assessing experienced utility in the sense used by Kahneman are criticized by economists, as they do not require the respondent to make a sacrifice. In other words, there is no opportunity cost, so there are questions about the meaning behind the expression of feelings, whether they can be compared between individuals, and whether they provide cardinal values.

Kahneman’s use of the term experienced utility can be compared with direct utility assessment, or patient own health state valuation, in the health economics literature. Patients own health state values can be obtained using visual analog scales (VAS), which may be considered a proxy for current momentary experience, but where those values are based on TTO, SG, or another choice exercise, it is a preference expressed over hypothetical future experience and a measure of decision’ rather than experienced utility in the way Kahneman has coined the term. The use of choice-based methods such as TTO to elicit values for experience-based utility requires respondents to value their current state by imagining what it would be like to be in full health for a shorter number of years—a state they may not have experienced for many years. For people who have lived in a chronic health state, e.g., chronic obstructive pulmonary disease or osteoarthritis, the task of imagining full health is potentially as difficult as a healthy member of the general population trying to imagine a poor health state. In addition, such individuals are being asked to imagine that they will remain in their current state for some specified period, such as 10 years in the case of the EQ-5D valuation study by Burstrom et al. [7], and then to imagine dying after that period. This is a hypothetical task and raises issues about how realistic this would be for most respondents and Kahneman’s question about people’s ability to predict their experienced utility into the future. Many concerns raised with the TTO task for hypothetical states are applicable to the TTO task for own health state valuation. For example, during the TTO task, do respondents really imagine their current health state will not change or do they imagine some change over time? Do they consider the fact they may adapt, that their life circumstances may change and so forth? Do they include more than the EQ-5D state they have just completed [e.g., their wider QoL (QoL), and if so, what else do they take into account? How do they deal with the idea of dying? SG addresses the concerns raised above with the addition that responses would also be impacted by respondents’ attitude to and perception of risk. Currently, we do not know the impact of all of these factors, and there is a role for qualitative research to examine what is actually being valued with these tasks. However, what we can conclude is that own health state valuation using TTO or SG is not the same as experienced utility in the way Kahneman and colleagues describe it. For this reason, throughout the remainder of the paper, we refer to own health state valuation rather than experience-based utility.

VAS has the advantage that it can arguably be viewed as a proxy for current momentary experience and so avoid some of the problems associated with choice-based tasks. It also has the ability to measure the value of the person’s own state where this can incorporate a wider notion of QoL rather than simply health. However, there are well-documented problems with VAS [1], including the use of rounded numbers (such as 80, 85, 90) rather than using all points along the scale and that the value may not represent utility, as it involves no sacrifice or tradeoff. An additional complication is that the value is not anchored on the 1–0 full health–dead scale unless there is some consideration of value of the own health state relative to the value for dead.

## Whose values matter

Chosing from whom to elicit values is important, because it may influence the resulting values. A number of empirical studies have been conducted that indicate that people with first-hand experience tend to (although not always) place higher values on dysfunctional health states than members of the general population who do not have similar experiences, and the extent of this discrepancy tends to be much stronger when people value their own health state [16–18]. However, there is some evidence suggesting that for mental health, values may be lower for people with experience of mental health problems [19] as, contrary to the general population, they tend to place a greater weight on mental health impairments relative to physical health impairments [20]. However, this research was based on valuing hypothetical states and not on valuing own health.

Earlier empirical studies comparing patient and general population values tended to use relatively small sample sizes and focused on differences in patient and general population values for a single medical condition or type of health problem. More recently, studies have attempted to use larger sample sizes across the general population or multiple patient samples, to compare across cells 1 and 4 of Table 1, and to collect large samples of data for cell 3. Mann et al. [5], e.g., estimated EQ-5D preference weights using regression analysis of own VAS data (cell 4) from patients diagnosed with eight different conditions (*n* = 3376) and compared results with EQ-5D preference weights estimated using regression analysis of hypothetical VAS values elicited from the general population (cell 1) (*n* = 2997; the measuring and valuing health (MVH) data set [21]). Compared with the general population, model the decrements for anxiety/depression were statistically significantly larger in the patient model but smaller for pain/discomfort and mobility. The magnitude of disagreement between the patient self-rated and the population VAS models was found to vary depending upon the patient’s condition. Rand-Hendriksen et al. [22] examined differences in the relative importance attributed to EQ-5D dimensions between own health state valuations from patients (*n* = 74,277) (cell 4) and hypothetical health valuations from the general population (*n* = 3773) (cell 1) for EQ-5D states using VAS in the United States. Self-care and pain/discomfort were the most important dimensions for the hypothetical health valuations, whereas usual activities was the most important dimension for own health state valuations. Little et al. [23] compared German own health state valuations (*n* = 2032, obtained from Leidl and Reitmeir [24]) and European hypothetical health valuations from the general population (*n* = 6870) (cell 1 vs cell 4) for EQ-5D using VAS, finding that pain/discomfort was the most important dimension for the own health state valuations. Sun et al. [6] generated EQ-5D preference weights by modeling own VAS using Chinese experience-based data (*n* = 120,709) (cell 3) and showed anxiety/depression had the greatest impact on own VAS values.

Burstrom et al. [7] used TTO (along with VAS) to estimate Swedish own health state value sets for EQ-5D-3L using general population health survey data (cell 3). A large sample (*n* = 45,000 individuals) was used to facilitate modeling of own health state TTO and VAS values in terms of the EQ-5D descriptive system. They found the anxiety/depression dimension had the greatest impact on both own TTO and VAS values.

In summary, although findings vary between these individual studies in terms of relative impact of dimensions according to own health state valuation, the available evidence highlights the potential for systematic differences between hypothetical general population preferences and own health state values that could impact results of an economic evaluation. The nature of this impact is examined.

## Why is there a difference between own and hypothetical values?

There are a number of possible contributing factors for observed differences between patient and general population values. Earlier, we argued that respondents in poor health states may find it hard to imagine full health. The literature suggests additional possible explanations, including poor descriptions of health states (for the general population), use of different internal standards, and adaptation or response shift [1, 3]. These are discussed in detail below.

### Poor descriptions of health states

An important potential source of discrepancy is found when descriptions provided to the general population in cell 1 may not accurately describe the health state, even when these are produced using a health state descriptive system. Respondents can bring their own information to the valuation exercise by drawing upon their own personal experiences or limited knowledge. Given that the personal experiences of people with a health condition and members of the general public are unlikely to be the same, it may mean that, in effect, they are evaluating different health states, even when provided with identical descriptions of the state to be valued. For example, anxiety and depression can be difficult to imagine if you have never experienced them, and no further clarification is provided in the description of an EQ-5D state. Patient respondents in cell 4 will not necessarily be valuing the health state they are categorized into by a descriptive system (like EQ-5D). Respondents are asked to value their current health state as they see it, and this may cover different dimensions to the measure being used. For example, there is some evidence that patients may interpret item response options differently: One patient with spinal cord injury answering a question on walking stated, “When I saw walking I just kind of took it as wheeling,” suggesting that walking was interpreted as the ability to get about [25]. Furthermore, it has been suggested that general population respondents in cell 1 focus too much on ill health and ignore the remaining positive aspects of a person’s life [3]. For example, the general population focusses on the negative aspects on a health state, whereas patients focus on both the positive and negative aspects [26].

### Changing internal standards

A well-known phenomenon in the psychometric literature is response shift, which refers to the possibility that individuals will change their internal standards for evaluating their own health in response to changes in their health [27]. Response shift occurs due to changes in expectations. For example, an older person may rate his or her health according to their expectations of the best possible health for a person of their age rather than best possible health per se. Similarly, a patient may rate his or her health by comparing themselves with other patients rather than with healthy individuals. In either instance, response shift will contribute to discrepancies between patient and general population values in cells 1 and 4 for the same health states and, unlike the problem of incomplete or inaccurate health state descriptions, it is difficult to see how response shifts can be reduced or eliminated in practice. Indeed, it can be argued that response shift effects in health state valuation tasks conducted with patients should not be of concern, since these reflect aspects of adaptation and coping.

### Adaptation to the state

Someone in a permanent and stable impaired health state is, depending on the health problem, likely to adapt over time, both physically and psychologically. Physical changes include acquisition of new skills to help cope with a disability, such as learning to use a walking stick. Or, a person may change the things they do to limit the impact of their disability or illness. For example, someone who once played football may take up a sport that has a lower impact on their knees. There are also psychological adaptations that include a shift in the relative weight that people place on different aspects of health and QoL and, more fundamentally, a change in their view of what matters in life. In addition, people may lower their expectations of what they can achieve.

It is well established in the literature that people tend to underpredict their ability to adapt to physical health conditions or impaired states [9, 28, 29]. When general population respondents read the description of a state, their valuation may reflect a response to, say, going blind, rather than being blind for an extended period. In other words, the general population focusses on the transition to the state rather than the longer-term consequences, and this results in the general population giving lower values compared with patient self-reported values for chronic states of health. The implications of this are examined below. For further discussion of normative arguments around the use of values that take into account adaptation to inform resource allocation decisions, see [9, 28, 29].

These explanations for the differences in values between the general public imagining the state (cell 1) and respondents valuing their own state (cell 4) may have implications, which are also examined below.

## How to collect own health state values

Asking respondents to value their own health state and to do so across a sufficiently wide range of health states of different types and severity raises two major practical problems. First, respondents in poor health states may be unable or unwilling to undertake complex and quite intrusive valuation tasks. This may be due to their physical limitations. Furthermore, there are also mental health and cognitive problems that make completing a health state valuation task (e.g., TTO) more challenging than simply completing the EQ-5D classification system, particularly if it is to be self-completed (which tends to be the case with many of these surveys). Furthermore, these tasks are completed only once by a respondent, without any assistance from interviewers and without a practice question (which is difficult for own health valuation). Second, there will be ethical concerns with asking people in terminal or incurable conditions to imagine hypothetical scenarios involving return to full health, accompanied by either the risk of immediate death or shortening life. For this reason, it is not possible to ask some patient groups to complete health state valuation tasks for any state (hypothetical or own). Combined, these two practical problems will result in lower numbers in the poorer health states being sampled, as shown by the low numbers reporting more severe levels of EQ-5D in Table 2 (taken from [6, 7]).

**Table 2:** Observed distribution of EuroQOL five dimensions questionnaire (EQ-5D) own health in surveys used to provide experience-based utility data

EQ-5D dimension	Level	Sweden (%) (*n* = 46,169) [7]	China (%) (*n* = 120,709) [6]
Mobility	1	90.1	94.8
	2	9.8	4.8
	3	0.1	0.4
Self-care	1	98.4	96.8
	2	1.2	2.8
	3	0.4	0.4
Usual activities	1	91.2	95.2
	2	7.7	4.0
	3	1.1	0.8
Pain/discomfort	1	50.8	90.8
	2	45.1	8.8
	3	4.1	0.4
Anxiety/depression	1	66.5	93.6
	2	30.8	6.0
	3	2.7	0.4

The data may also be suceptible to selection bias, since people experiencing a health state that has impacted more on their utility may not wish to participate in elicitation surveys (even if they are able to). The magnitude of this type of selection bias is likely to vary not only by the severity of the condition but also by dimension (e.g., mental health). This will introduce a complex pattern of bias, with some (but not necessarily all) severe states having higher values than would be the case if it was a genuine random sample of people in those particular states. This may partly explain why own health state values are higher than the values of members of the general public imagining them. A major practical problem for the researcher is how to obtain a sample that is representative across the severity range. This is a survey design issue, and a different approach is proposed in below. Of course, it is also recognized that if some people in poorer health are less likely to participate in surveys, this impacts on the representativeness of results for both own state valuation surveys and surveys valuing hypothetical states. However, it could be argued that for evaluating hypothetical states, any impact would arguably be across all health states and would not be differential across different severity of states or across dimensions, meaning that for policy purposes, the incremental QALY change across interventions would not be expected to be impacted.

### Econometric issues

The elicitation of own health state values for EQ-5D in the literature has involved the use of regression analyses of large general population samples who value their own health using TTO [7] or VAS [6, 22, 24] to produce preference weights for every health state defined by the classification system. Sample sizes for these studies were large: 49,169 for Sweden [7], 74,277 for the USA [22], and 120,709 for China [6]. However, as these were general population samples, most respondents were in full or mild health states. Table 2 reproduces the distribution of (three-level) EQ-5D responses in the Swedish and Chinese samples used to estimate preference weights for the EQ-5D based on own health state values. In both samples, the proportion of responses in level 1 in each dimension is large, and 39.8 and 87% of respondents are in EQ-5D full health in the Swedish and Chinese samples, respectively. However, the distribution for Sweden has larger proportions of respondents at levels 2 and 3 for pain/discomfort and anxiety/depression dimensions.

The distribution of EQ-5D responses with most responses at level 1 and a small proportion of responses at the lowest level 3 means there is a large number of observations with TTO or VAS data for mild health states and only a small number for severe health states. This creates problems for modelling data to generate preference weights for every possible health state defined by the classification system. Even in the hypothetical health state valuation literature, it is usually not feasible to value all health states defined by a health state classification. Health states for hypothetical valuation are usually selected by design, such as an orthogonal array (e.g., [30]), balance (e.g., [31]), or simulation of alternative selections (e.g., [32]. All of these approaches select health states to ensure that the model estimated using the preference data is able to produce preference weights with acceptable margins of error for every possible health state defined by the classification system, and thus selecting health states is an important component of any health state valuation study. However, the approach used in own health state valuation does not select health states in any systematic way. Also, it does not necessarily include any data on many of the health states, as 148 and 167 unique health states were observed in the Swedish and Chinese samples, respectively, out of the 243 possible EQ-5D states. It is likely that this will detrimentally impact on the accuracy of any model used to estimate values for every health state defined by the classification system.

It may be argued that while efficient designs aim to spread the prediction error across dimension levels and thus across states, own valuation data sets contain more data on precisely those states for which information it is needed, i.e., the most frequent states. At the extreme, there is no need to predict the value of health states that do not exist in the real world with the same (or even any) accuracy as those states that occur more often. However, states that drive the results of cost-effectiveness models may not be the ones that arise with sufficient frequency in a general population sample. Cost-effectiveness models are based on conditions and for different stages of disease progression across different treatments, rather than EQ-5D health states per se, making it difficult to determine which EQ-5D health states are used in practice to estimate utility values for cost-effectiveness models across a wide range of patient groups and interventions.

### A way forward: How could own valuation studies be designed?

An alternative approach to large-scale general-population own health state valuation surveys is to purposively sample people to provide own TTO values to ensure that values are obtained for health states that are informative for estimating a regression model estimating preference weights for every state described by the descriptive system. For example, a set of health states for, say, the EQ-5D-5L could be selected using a statistical design, and quotas could then be set for, say, 200 respondents in each health state and respondents from a sampling frame ensuring that respondents are representative of that state in terms of sociodemographic characteristics, such as age and gender (which usually will not be the same as for the general population).

To give an idea of the numbers involved, let us examine the numbers required in a general population survey using our experience from three online general population surveys. In the pool of 8600 respondents across the three surveys [33, 34], we observed 586 of 3125 unique EQ-5D-5L states broken down as follows: 11,111 had a 35% share, the next two states covered another 20% of respondents, and the next 20 states covered another 30%. Only the top six states had an > 200. The 200th ranked state had just three observations. This would suggest that most EQ-5D-5L states (> 90% of them) have a prevalence rate of ≤ 0.02% in the general population. This suggests that to identify 200 individuals in a health state with a 0.02% prevalence, this would require screening one million individuals to find them (and not all of them may agree to be surveyed). There are many assumptions in this rough calculation that we would not care to defend, but a crucial one is that an online survey using self-completed health state valuation tasks of own states is likely to suffer from the biases raised earlier. To achieve representative samples in the more severe states requires more directed sampling strategies and different modes of administration to ensure the more disabled or dissatisfied are not excluded.

The advantage of taking a designed approach is that data would be informative for estimating regression coefficients to produce weights for all health states defined by the descriptive system with acceptable levels of error, and values for these would be for own health, not hypothetical health, states. However, the challenges of finding people in more severe health states would be considerable, and the design would need to take into account the plausibility and prevalence of the more severe states selected in the study. Also note that such a sample that extensively oversampled respondents in low-prevalent states would no longer be a representative sample, where all members of the general public had an equal chance of being recruited.

## Why use (or why not use) own health state values?

The original Washington Panel on the Cost Effectiveness in Health and Medicine, published in 1996 [35], advocated the use of general population values and argued that: “… the best articulation of society’s preferences for a particular state would be gathered from a representative sample of fully informed members of the community” (this has been reaffirmed for the reference case in the update [36]). The panel went on to use the notion of the veil of ignorance to support the use of community values, where: “a rational public decides what the best course of action is when blind to its own self-interest, where aggregating the utilities of persons who have no vested interest in particular health states seems most appropriate” [35]. Swedish guidelines for health technology assessment (HTA) from the Pharmaceutical Benefits Board state that utilities should be generated using patient values and own health state valuation: “Quality-adjusted life-year (QALY) weightings should be based on methods such as the SG or TTO methods. QALY weightings based on appraisals of persons in the health condition in question are preferred before weightings calculated from an average of a population estimating a condition depicted for it” [37: p2]. However, many other international agencies providing guidelines for their preferred methods for HTA submissions prefer hypothetical values elicited from the general population (Australia [38], Canada [39], France [40], The Netherlands [41, 42], Spain [43], UK (England and Wales [44], Scotland) [45]), though Australia, Canada, and Scotland accept own health state utility values (see [46] for an overview of international recommendations and regulations for utility data for HTA). People tend to compare themselves to peers with similar health problems, which will influence their own health state valuations. They argue that the values of different patient groups are not comparable, whereas a general population sample provides a coherent set of values.

A key argument is that the general population pays for the service. However, whereas members of the general population may want to be involved in healthcare decision making, it is not clear that they want to be asked to value health states specifically (see, e.g., [47]). At the very least, it does not necessarily imply that the current practice of using relatively uninformed general population preferences is optimal. An argument for using own health state values is that people understand the impact of their health on their well-being better than someone trying to imagine it (although they are having to imagine full health in most health state valuation tasks). Nevertheless, this does not necessarily imply that own health state values should be used on their own to inform resource allocation decisions. This requires a value judgement that society wants to incorporate all the changes and adaptations that occur in people who experience states of ill health over long periods of time. Some adaptation may be regarded as laudable, such as skill enhancement and activity adjustment, whereas cognitive denial of functional health, suppressed recognition of full health, and lowered expectations may be seen as less desirable [2]. Furthermore, there may be a concern that own health state values are context based, reflecting comparisons with their recent experiences of ill health and the health of their immediate peers [3], which relates to a response shift, as discussed above.

## Alternative approaches

One conclusion from the above discussion is that it may be difficult to justify the exclusive use of own health state values or the currently widely adopted practice of using values from relatively uninformed members of the general population. If it is accepted that, ultimately, the values of the general population are required to inform resource allocation in a public system, it can be argued that respondents should be provided with more information on what the states are like for people experiencing them so they can provide informed values. There are many different ways of achieving this objective:

1. Improve the descriptive systems, e.g., include well-being dimensions that better reflect the impact on the lives of those experiencing the health states.
2. Encourage more deliberation and reflection in the task.
3. As in (2), but provide more information on adaptation or own health state values (e.g., through own TTO values for people in those health states) to the general population sample undertaking the valuations.
4. Provide decision makers with two incremental cost-effectiveness ratios or net benefit values using (a) general population hypothetical values and (b) own health state values [4].
5. Use subjective well-being to reweight an existing health state classification system, such as the EQ-5D [48].

Option 4 would add significantly to decision complexity and would imply the need for two threshold values. Unless the two analyses support the same decision, it further begs the question of how a decision maker decides which analysis or weights to use if they were to be combined. It could result in inconsistencies across decisions that would be difficult to defend, particularly when decisions are appealed in a court of law. We argue that it is better to agree on a single value set for decision making. Option 5 presents the challenge that results are not anchored onto the 1–0 full health–dead scale required to generate QALYs, as currently, no measures of subjective well-being have been valued using this scale. Below, we focus our discussion on options 1, 2, and 3.

### Improving the descriptive system to take better account of the impact on well-being

The description of health states used in valuation surveys relies on instruments like the EQ-5D, which covers the dimensions of mobility, self-care, usual activities, pain/discomfort, and anxiety/depression. This may be too narrow for some areas of health care [49] and does provides little information to general population respondents on the likely impact of these health problems on their lives. One solution to this problem is to develop a broader QoL measure, which describes the impact on functioning and well-being, and this option is being explored in the E-QALY project being undertaken by a number of the authors (JB, DR, TP, AT) [50]. This new generic measure may reduce the gap between general population hypothetical values and own valuations, since it provides a broader description of the impact of a health state.

### Encouraging more deliberation and reflection using conventional valuation methods

Encouraging more deliberation and reflection has been proposed by a number of commentators on the subject [2, 3, 28, 29, 51], and there are examples of some studies attempting to operationalize this in practice. A review of empirical studies attempting to inform general population respondents, published in 2009 and earlier, identified 14 studies reporting on methods used to elicit informed general population values for health states [52]. Interventions were categorized into the following: information to enrich health state descriptions (*n* = 7); simulation to reproduce symptoms of the health state (*n* = 2); opportunity to reflect and deliberate on health state descriptions (*n* = 2); exercises to evoke adaptation to the health state (*n* = 3). Most studies identified in the review attempted to generate informed general population values for health states by providing respondents with additional information using audio recordings and videos. These studies appear to show that general population values can be changed by providing additional data, though it is difficult to judge whether the population was better informed other than the finding that values were closer to patient values. For example, Clarke et al. [53] examined health state values for three Gaucher disease states by presenting information from patients currently living in the states. The authors used multimedia equipment, finding no statistically significant differences in utility between patients and general population samples. Cunningham and Hunt [54] used descriptions of dentofacial deformities and photographs of dental patients corresponding to the health state descriptions, again finding no statistically significant differences in utility between patients and general population samples. Damschroder et al. [55] used an adaptation exercise when valuing states pertaining to pre-existing and new-onset paraplegia, finding that completing an adaptation exercise statistically significantly increased utility values. McTaggart-Cowan et al. [28] explored the extent to which members of the general population changed their initial values for three rheumatoid arthritis states following an adaptation exercise; respondents listened to recordings of patients discussing how they adapted. After undergoing the adaptation exercise, respondents increased their values for rheumatoid arthritis states, and younger and healthier individuals were more likely to increase their initial values after being informed.

However, one study found that discussion of preferences elicited in a group setting did not have a statistically significant impact on responses. Stein et al. [56] used SG with a panel of 15 members of the general population who valued 41 different health states five times over the course of 6 months. Following initial individual valuations, the group was given the opportunity to discuss the health states, and following this discussion, individuals were given the opportunity to change their health state values, although with no reference to patient values or adaptation. Although no statistically significant differences were detected before and after the discussion, respondents indicated that the group discussion brought reassurance and cohesion to their responses.

Overall, this evidence suggests that informed general population preferences can reduce the difference between own health state values, though whether the values are therefore better informed is difficult to prove, and further research is encouraged [28, 57, 58]. The previous studies were concerned with small numbers of mainly condition-specific states. It is not clear how practical these methods would be for valuing large numbers of generic states generated by measures such as the EQ-5D-5L. There is also concern that the impact on health state values would differ depending on the condition related to the underlying health state, adaptation to that health state, and the impact of that condition. For example, the impact on health state values from adaptation to problems with self-care may differ depending upon whether the problems were caused by rheumatoid arthritis, Parkinson’s disease, or stroke. One option is to not identify the condition but, instead, to widen the impact of the health state on wider QoL and non-health-related consequences, such as enjoyment, independence, relationships, and dignity [59]. This is one option being explored by the authors through the development of a broader descriptive system (i.e., option 1 above).

### Using citizens’ juries and MCDA

Citizens’ jury and multicriteria decision analysis (MCDA) can also be used to elicit informed utility values from the general population. However, as far as we are aware, they have not been used to value health states, and the methods may understandably need further consideration and adaptation to the context. A citizens’ jury involves a small sample of participants deliberating on a topic and reaching a democratic recommendation [60]. In the area of health policy, a sample of general population participants: (a) are presented with a health policy dilemma, (b) review and examine evidence on the dilemma, with the presentation of the evidence undertaken by experts, and (c) deliberate the dilemma to reach a consensus for a recommendation, which not all participants have to agree with [60]. Participants in a citizens’ jury are not typically expected to be informed prior to the process; rather, they become informed during the process. The National Institute for Health and Clinical Excellence (NICE) Citizens’ Council is an example of a citizens’ jury that is routinely involved in health-policy decision making (though this is just one type of citizens’ jury). The outcome of the citizens’ jury is typically qualitative, meaning that the strength of preference is not typically indicated. Therefore, a citizens’ jury would need to be combined with one of the other techniques, such as TTO. This could involve participants undertaking TTO tasks “cold” before becoming informed on the topic, undertaking the full deliberative process of the citizens’ jury, and then completing the same TTO survey again to provide informed responses (see, e.g., [61]). Undertaking the same TTO survey before and after the deliberative process enables the impact of the deliberative process to be assessed.

MCDA can be used to evaluate health states in a group setting by explicitly considering multiple criteria. This enables a structured approach as:

1. Criteria are determined
2. Scores reflecting the value of how health states perform according to each multiple criteria item are determined
3. Weights that reflect the relative importance of each different criterion are considered
4. A recommendation is reached [62].

Different types of approaches can be used to score and weight criteria: value measurement models; outranking models; goal, aspiration, or reference-level models [62]. Scores and weights can be determined separately, e.g., using VAS; or scores and weights can be determined simultaneously, e.g., using discrete choice experiments or TTO (see [63]). This could be similar to recent work on “personal utility functions” undertaken by Shah et al. [64].

## Conclusions

This paper has critically examined the recent interest in the use of experience-based utility and own health state valuation for existing health state classification systems. We conclude that TTO and VAS tasks do not measure experience utility, as described by Kahneman and colleagues, and we refer to own health state valuation rather than experience-based utility. The literature has reported on the collection of large amounts of quite complex data from people who may not be actually valuing what we imagine they are valuing and not providing values of health states across the severity range. Though arguments for eliciting own health state values require consideration, the suitability of current preference weights generated using own health state values for informing policy should be questioned.

We proposed one approach for collecting own health state values that would provide less biased data for modelling values to produce preference weights by systematically selecting health states and purposively sampling respondents using a sampling frame to meet predefined quotas. However, this does not address the issue that the valuation task would still most likely need to be self-completed only once, without any assistance from interviewers or a practice question, which is likely to impact data quality. Other possible methods of eliciting own health state utility values that do not have these problems would be of great value. Finally, we suggested alternative approaches of obtaining more informed general population preferences that take more account of patient views and experience. This is a promising way forward that warrants further exploration.
